# Intermittent Active Inference

**DOI:** 10.3390/e28030269

**Published:** 2026-02-28

**Authors:** Markus Klar, Sebastian Stein, Fraser Paterson, John H. Williamson, Henrik Gollee, Roderick Murray-Smith

**Affiliations:** 1 School of Computing Science, University of Glasgow, Glasgow G12 8RZ, UK; sebastian.stein@glasgow.ac.uk (S.S.); johnh.williamson@glasgow.ac.uk (J.H.W.); roderick.murray-smith@glasgow.ac.uk (R.M.-S.); 2 James Watt School of Engineering, University of Glasgow, Glasgow G12 8QQ, UK; henrik.gollee@glasgow.ac.uk

**Keywords:** active inference, intermittent control, free-energy-principle, computational efficiency, human-computer interaction, resource-aware algorithms, mouse pointing

## Abstract

Active inference provides a unified framework for perception and action as processes of minimizing prediction error given a generative model of the environment. Whilst standard formulations assume continuous inference and control, empirical evidence indicates that humans update their control strategies intermittently, which reduces computational demands and mitigates propagation of correlated noise in closed feedback loops. To address this, we introduce Intermittent Active Inference (IAIF), a novel variant in which sensing, inference, planning, or acting can occur intermittently. This paper investigates intermittent planning, where IAIF agents follow their current plan and only re-plan when the prediction error exceeds a predefined threshold or the Expected Free Energy associated with the current plan surpasses prior estimates. We evaluate intermittent planning in a mouse pointing task, comparing against continuous planning while examining the impact of different threshold parameters on performance and efficiency. The findings indicate that IAIF reduces computation time whilst maintaining task performance, particularly when the number of plans sampled during planning is increased. In case of the proposed trigger based on Expected Free Energy, no additional calibration is required for this. The straightforward integration of IAIF makes it valuable in practical modelling workflows.

## 1. Introduction

Computational models of agentic behavior are commonly studied in the framework of Markov Decision Processes (MDPs), where agents and their environments update action states and observation states, respectively, once per discrete time interval. These intervals are commonly of constant duration, which is often set conservatively short to ensure that high-frequency dynamics are represented sufficiently accurately within successive state updates [1]. Active Inference (AIF) agents simulate a number of internal cognitive processes in order to determine a plan of successive future actions. By cognitive processes we mean updating their internal beliefs about the hidden state of the environment from new observations; updating their internal beliefs about the hidden state of the environment after updating their action states; updating their internal model of the environment from new observations; and updating the sequence of successive future actions. Standard formulations of AIF agents assume that all internal cognitive processes are executed at the same temporal frequency, i.e., once per MDP timestep [2]. It is not clear what trade-offs are associated with this design choice regarding system identification (learning) convergence, state inference, computational efficiency, and behavioural performance. In particular, as AIF is increasingly being taken up as a paradigm within agent-based artificial intelligence and autonomous robotics [3,4,5], addressing the computational difficulties associated with inference and planning constitutes a major practical concern.

In another line of research, elements of human motor control have been shown to be more consistent with an intermittent control model, where open-loop “ballistic” movements are updated intermittently based on event-triggers and refractory periods, than with continuous control or strictly time-triggered control [6]. These models exhibit computational advantages and robustness of system identification to delays, noise and constraints. In Reinforcement Learning (RL), temporal abstraction through generalized actions (options) and semi-MDPs [7] have shown to speed up planning and learning. In networked systems, event-triggered control significantly reduces communication bandwidth and computational resources [8]. Clearly, for any agent with constrained computational resources it would be prudent to decide when and how frequently to execute individual cognitive processes based on their expected contribution to maximising utility [9] or minimising surprise.

**Figure 1:**
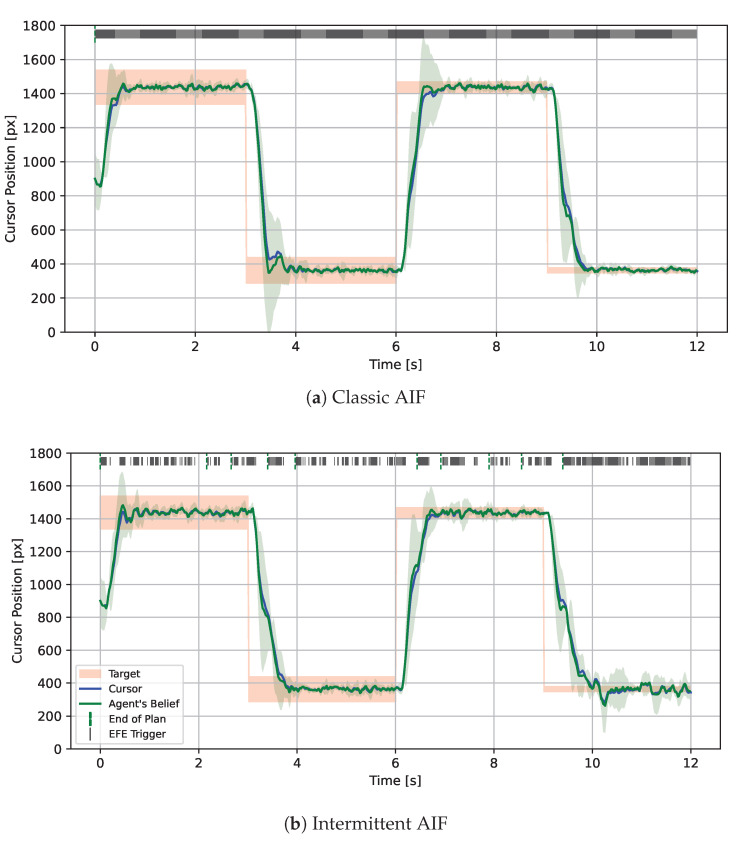
**1D Mouse Pointing Task simulated with Classic and Intermittent Active Inference (IAIF) for Different Target Sizes.** The blue line shows the true cursor position while the mean of the agent’s prediction is displayed as a green line together with a 3σ ribbon. The targets are shown as red areas. Every time step in which the agent plans is marked by a solid line at the top of the plot. (**a**) The classic AIF agent observes and plans at every time step, leading to high computational cost. In particular for larger targets, the agent could follow a plan for longer without leaving the target area. (**b**) This intermittent AIF agent only starts a planning phase when the EFE of the current plan turns out to be worse than predicted during planning (indicated by a black line on the top ‘EFE Trigger’). This primarily happens when the agent observes a target change or when the cursor gets close to a new target. As soon as the cursor reaches a target and slows down, the uncertainty of the agent’s prediction (green area) is reduced. In this phase, the agent only plans intermittently. Since accidentally moving out of the target becomes more likely for smaller targets, the frequency of re-planning events increases. The agent will also start a new planning phase if it reaches the end of its planning horizon, indicated with green lines at the top of the plot.

In this paper, we introduce Intermittent Active Inference (IAIF), taking inspiration from Intermittent Control to propose event-triggered, intermittent perception, inference, system identification, and planning. We have previously proposed that Active Inference could provide a useful basis for Human-Computer Interaction (HCI) research and design in [10]. So, we now explore intermittent planning on a mouse pointing task and present quantitative evidence from computational simulations that characterizes the effect of triggering mechanisms and associated thresholds on computational efficiency and on task performance. In order to simulate agent behaviour, we build on a recently presented AIF agent for systems with continuous state, observations, and actions [11]. Interestingly, we observed consistent task performance at reduced computational cost compared to continuous re-planning and identified that this is caused by sample-based, approximate rollout evaluation. Figure 1 shows mouse cursor trajectories for classic and intermittent AIF side by side. The IAIF agent only starts a planning phase when the current plan becomes worse than expected or ends. This frees computation, in particular for larger targets. For smaller targets, frequent re-planning is required to ensure that the cursor stays inside the target. We provide the JAX-based (https://docs.jax.dev/en/latest/index.html, accessed on 5 February 2026) implementation of IAIF and code to run the simulations performed in this work on GitHub (https://github.com/mkl4r/iaif, accessed on 27 February 2026).

### Contributions

The primary contributions of this work to the Active Inference and Human-Computer Interaction communities include:

- introducing Intermittent Active Inference as a simple extension of classic AIF with the potential to reduce computation time and improve the realism of human motor control simulations,
- evaluating two trigger mechanisms for intermittent planning and their combination against a standard AIF agent in a 1D mouse pointing task—a classic HCI problem,
- discussing further implications of intermittency in AIF, and,
- providing Python (version 3.12.12) code (https://www.python.org/) to simulate IAIF agents for continuous control tasks with perceptual noise and delay.

## 2. Related Work

### 2.1. Continuous Active Inference

Active Inference is a closed-loop computational theory for modelling agent behaviour [2,12]. Many of the introductory examples in the research literature have involved discretisation of the state space for computational convenience. Other approaches to manage the challenges of continuous spaces include: (1) learning latent embeddings, (2) hierarchical models, (3) habitual control and (4) event-driven/intermittent approaches:

(1) Matsumoto et al. [13,14,15] inferred low-dimensional latent variables to perform efficient goal-directed plan search in continuous action spaces. Hybrid approaches are presented in [16], and applied to the continuous mountain car problem. (2) Using hierarchical generative models allows agents to plan at multiple temporal and spatial scales, which has the potential to alleviate the problems associated with frequent re-planning [2]. (3) Habitual control behaviours have been used in AIF to reduce the computational cost of prediction-based control in contexts which are well-understood by the agent [17]. The agent relies on prior beliefs over policies, and these priors encode how often past policies have succeeded. This creates a bias toward well-practiced, familiar actions, even when they are not fully evaluated by predictive simulation in the current context. An AIF agent can be given a mechanism to ‘cache’ policy probabilities from previous trials, and reuse them to reduce the inferential steps of deliberative processing [18]. In [19], a meta-cognitive control level is added to switch between habitual controllers and predictive controllers if the context changes. (4) The Bayesian inference suite RxInfer includes agents that exhibit event-driven updates in perceptual inference, but not yet in planning, policy selection and action execution [20]. To our knowledge, single-agent AIF systems that directly employ intermittency in control have not been explored.

These issues are not only relevant to AIF systems, so AIF research can learn from other areas such as (a) RL and (b) Model Predictive Control (MPC) systems: (a) When to re-plan is critical in model-based RL. Honda et al. [21] observe that *‘Too much re-planning could also cause path oscillation, especially when sampling-based global planners are utilized or the environment has many branching pathways.’*, which is the case in continuous-action AIF. (b) Plan consistency between samples is a key challenge in sample-based MPC, as solutions from different optimization runs or samples can be highly variable, leading to jerky or inconsistent control actions [22]. Sampling methods are often iterative local searches that do not guarantee convergence to the global optimum or a consistent local optimum across different sampling iterations, especially in high-dimensional problems or when computational issues limit the sample size available. Methods such as warm-starting, using the optimal trajectory from the previous step can significantly reduce variance and computation time, and improves consistency across time steps. Ref. [23] is an example of a link being made between MPC and active inference, by using EFE as an objective function for MPC.

### 2.2. Human-Computer Interaction

As Martín et al. [24] observed, since the advent of graphical user interfaces, aimed movements towards a spatially defined target have become a primary means of input to computers. Such movement during interaction with computers is inherently dynamic and happens in a feedback loop. Users observe the current state of the computer (e.g., cursor position) and adjust their movements to change this state into the state they desire—“*movements only make sense when they are precisely located in time and space that is, when they are part of an action, seeking to accomplish a goal. In this sense, simple goal-directed movements, such as pointing and grasping, can be considered to be the building blocks of more complex actions*” [25]. The role of simulation in HCI has been growing recently [26], and simulation-based models of interaction that use MPC or RL combined with biomechanical models of the human upper extremity have been introduced [27,28]. These simulations yield full joint movement trajectories or muscle activations which enables tuning of interaction systems to improve performance and ergonomics.

#### 2.2.1. Submovements and Interaction

In HCI, movement is also often understood as a series of events, such as submovements, and the *Iterative Corrective Submovements* model proposed by Crossman and Goodeve [29] is widely used. This is a model where an aimed movement is understood as a series of individual submovements towards the target, each with a constant error and constant duration. Crossman and Goodeve show how Fitts’ law can be derived from this model. This includes well-known HCI models, such as the work by Card et al. [30], who introduced their ‘GOMS’ framework where a Model Human Processor represented movement as a series of discrete steps. Schmidt’s Law [31] addresses variability and dynamics by manipulating amplitude and movement time and measuring the effective target width $W_{e}$, leading to the relation $W_{e}=k\frac{W}{MT}.$ The insight behind Schmidt’s Law is that the human controls movement via discrete force impulses and the overall variability is from the variation of the magnitude and duration of the applied force. Meyer et al. [32,33] further developed this to the *optimised dual-submovement model* which is consistent with both Schmidt’s and Fitts’ laws, with the variability of submovements being proportional to the average velocity and that variability leading to the requirement of multiple sub-movements optimised to minimise the total movement time. Analysis of cursor pointing tasks in humans also found different stages of open-loop and closed-loop control [34]. These models included continuous second order dynamics models, and Costello’s surge model, which has a switching characteristic [35].

#### 2.2.2. Human Motor Control

While human motor control is often approached as a continuous control problem with added noise [36], there is substantial evidence of a combination of open-loop and closed-loop processes in movement control, for example in the context of refractoriness [37] and limitations in the frequency content of human movement dynamics and variability [38].

### 2.3. Intermittent Control

Intermittent control (IC) uses feedback information intermittently to re-plan an open-loop control action if and when needed. Although various approaches to intermittent control exist (see [39] for an overview), re-planning events are usually triggered by a divergence of observed from predicted states [6]. IC originates from practical implementation of MPC in the presence of constraints [40]. Compared to continuous feedback control, where the control action is recalculated at each time point based on observations, intermittent control reduces the overall bandwidth, freeing up resources for optimisation tasks [6].

IC offers a physiologically plausible explanation for non-linear and non-continuous observations in human balance control and other motor tasks [41,42]. A general description of motor control includes sub-cortical continuous feedback elements which are associated with fast reflex actions, combined with slower, intermittent feedback loops which involve cortical processing and are gated by the basal ganglia [43,44].

Using feedback information only intermittently to modify the control plan instead of continuously updating the control action has also been shown to facilitate parameter estimation and system identification in a closed-loop configuration in the presence of noise [45], where the propagation of correlated noise is reduced as a result of the open-loop intervals.

## 3. Materials and Methods

In Section 3.1, we will first introduce the methodical background, that is, continuous, sample-based AIF. This section does not contribute novel methodology and its main purpose is to introduce the general interaction loop between the AIF agent and the environment, and notation. Readers that are familiar with applied AIF may want to directly jump to Section 3.2 where we present Intermittent Active Inference and how to implement intermittent inference and intermittent planning. We then formulate two different approaches to triggering a planning phase based on belief divergence (Section 3.2.1) and Expected Free Energy error (Section 3.2.2). Finally, in Section 3.3, we describe the design of the 1D mouse pointing task and give details on the agents that are evaluated.

### 3.1. Active Inference for Models with Continuous State, Actions, and Observations

Active Inference offers a mathematical account of *agency* in terms of a process of approximate Bayesian inference [46]. The fundamental imperatives for an AIF agent consist in the necessity of predicting and anticipating the various dynamics of the environment in which the agent is situated. This process is ultimately in service to the agent’s autopoietic goal of realising the fulfilment of its preferences. AIF agents are embedded in an environment, which is mathematically accounted for in terms of a state space and its associated dynamics. Typically, the states are hidden states, in that they are not directly observable by the agent. The agent makes observations that are functions of the hidden states, on the basis of which the agent may then make inferences about the potential latent/hidden states that gave rise to a specific observation. Hence, AIF provides a probabilistic framework for modelling the perception-action loops that characterise an agent’s interaction with an environment.

In this study of a biomechanical cursor-pointing application we are concerned with the continuous state-space of forces, displacements and velocities. Hence, both the agent and its environment are described in terms of dynamical systems with continuous latent states, actions and observations. Each of these evolves in discrete-time.

We employ the term *generative process* to denote the environmental dynamics in which the agent is situated, as per the wider AIF literature [2]. In this work, we assume discrete time steps t∈N and that the generative process is characterised by deterministic state-transitions and that the hidden states afford stochastic observations to the agent. Hence, for continuous latent states s[t]∈S, control input/action a[t]∈A and observations o[t]∈O, the generative process takes the form:

(1) $$\begin{array}{cc} s[t+1] & =f_{\theta }\left(s[t],\;a[t]\right) \end{array}$$

(2) $$\begin{array}{cc} o[t+1] & ∼N\left(g\left(s\left[t+1\right]\right),\;\Sigma_{p}\right), \end{array}$$

where $\Sigma_{p}$ denotes the covariance matrix of the Gaussian observation noise. $f_{\theta }$ denotes the non-linear state-transition dynamics parametrised by θ. g(·) is the observation map, which generates the “raw” observation as a mapping of the hidden state. The actual value observed by the agent is the sampled (noisy) observation in Equation (2). Now the means by which an AIF agent actually makes inferences about hidden states (perception) and selects appropriate controls with which to steer the dynamics of the environment (action) consist in its modelof the generative process. This model is called the *generative model* (GM) [2] and is given by the dynamics and observation functions,

(3) $$\begin{array}{cc} \hat{s}\left[t+1\right] & ={\hat{f}}_{\hat{\theta }}\left(\hat{s}\left[t\right],\;a\left[t\right]\right)\;\mathrm{and} \end{array}$$

(4) $$\begin{array}{cc} \hat{o}\left[t+1\right] & ∼N\left(\hat{g}\left(\hat{s}\left[t+1\right]\right),\;{\hat{\Sigma }}_{p}\right). \end{array}$$

Equations (3) and (4) specify the structural form of the agent’s generative model; together with priors over hidden states, parameters, and noise variables, this system induces a joint probabilistic generative model (proper). Inference is performed by maintaining variational beliefs over the latent variables of this model. We have used “hatted” superscripts: $\hat{\cdot }$ to denote a component of the generative model, as distinct from the generative process. The GP and GM are separate dynamical systems. Importantly, the generative model need not coincide with the true generative process and typically constitutes a coarse-grained approximation of the GP. Hence, with the GM, the agent has an internal representation of the GP with which it can make predictions and form beliefs.

In Section 3.1.1, we will see that the agent maintains probabilistic beliefs over internal variables, expressed as distributions such as $Q^{s}$. For notational simplicity, we will subsequently write $Q^{s}$ to denote the agent’s belief about the external state, with the understanding that this belief is always, actually supported by the internal model variable.

Taken together, the GP and GM define a closed sensorimotorloop. Observations are generated by the environment (GP) and are assimilated by the agent—via inference—to update its beliefs about hidden states, parameters and noise variables. These beliefs are then used to evaluate candidate action sequences through predictive rollout under the GM, constituting planning and action selection. The selected action is applied to the GP, influencing its subsequent state transition and observation, thereby closing the perception-action cycle for the current time step. In classic AIF, inference and planning are performed in every time step. The belief update components of the sensorimotor loop are detailed in Section 3.1.1. The planning and action-selection mechanisms are likewise detailed in Section 3.1.2.

#### 3.1.1. Belief Update

We now sketch the mechanism of belief updating in AIF. In line with our previous work [11], we endow the agent with approximate posterior beliefs about the latent states: *s*, model parameters: θ and observation noise: $\Sigma_{p}$. We assume that this joint belief amounts to a mean-field factorisation of the approximate posterior:

(5) $$Q\left(s,\theta ,\Sigma_{p}\right)=Q\left(s\right)Q\left(\theta \right)Q\left(\Sigma_{p}\right).$$

To efficiently update the agent’s belief when performing an action, we apply an *Unscented Kalman Filter* (UKF) which propagates normal distributions through non-linear dynamics [47,48]. Hence, at time step t∈N the agent applies the action a[t] and updates its belief about the system states $Q^{s}\left[t\right]$, using its GM ${\hat{f}}_{\hat{\theta }}$ and belief about system parameters (such as target position) $Q^{\theta }$,

(6) $${\hat{Q}}^{s}\left[t+1\right]:=\mathrm{UKF}\left(Q^{s}\left[t\right],a\left[t\right];{\hat{f}}_{\hat{\theta }},Q^{\theta }\right).$$

Upon receiving a new observation from the environment, the agent would ideally update its prior belief over latent states according to Bayes’ rule:

(7) $$p\left(s|o\right)=\frac{p(o|s)p(s)}{p(o)}$$

In most applications, the dimensionality of the latent-state renders the marginalisation problem in the denominator of Equation (7) intractable. Instead of exact Bayesian inference, AIF employs *variational inference* (VI), in which an approximateposterior distribution q(s)≈p(s∣o) is chosen from among a specific family of distributions and optimised to approximate the true posterior. At time t∈N and for a given predicted belief ${\hat{Q}}^{s}\left[t\right]$ and observation o[t], we denote the VI step as

(8) $$Q^{s}\left[t\right]=\mathrm{VI}\left({\hat{Q}}^{s}\left[t\right],o\left[t\right]\right),$$

resulting in the agent’s updated belief $Q^{s}\left[t\right]$. In contrast to Generalised Filtering [49], the UKF and VI steps explicitly estimate the belief covariance. This tracks poorly controlled and more chaotic systems better but comes with additional computational cost.

#### 3.1.2. Planning Phase

In order to select a new plan, the agent first samples K∈N plans. For our purposes a ‘plan’ $\pi_{j}$ is a sequence of N∈N actions sampled from the agent’s action prior $P_{a}$, i.e., $\pi_{j}={\left(\pi_{j}\left[i\right]\right)}_{i}$, $\pi_{j}\left[i\right]∼P^{a}$ for i∈{1,…,N}, j∈{1,…,K}. Each action in a plan indexes the point in time after planning at which the action is to be executed. Hence, a plan with *N* actions is a sequence of temporally contiguous actions for each of the *N*—future—time steps. The agent then evaluates each sampled plan by ‘rolling it out’. The process of rolling out a given plan consists in the agent predicting changes in the environment, conditioned upon the execution of each action in the plan and the agent’s GM. This results in a sequence of *predicted beliefs* for all *N*—future—time steps ${\hat{Q}}^{s}\left[t+i\right]$ for i=1,…,N

(9) $${\hat{Q}}_{j}^{s}\left[t+i\right]:=\mathrm{UKF}\left({\hat{Q}}^{s}\left[t+i-1\right],\pi_{j}\left[i\right];{\hat{f}}_{\hat{\theta }},Q^{\theta }\right),\;{\hat{Q}}_{j}^{s}\left[t\right]=Q^{s}\left[t\right].$$

These roll outs are then evaluated with respect to their individual *Expected Free Energy (EFE)* $G(\pi_{j})$ which is the mean of the step-wise Expected Free Energies $G_{j}\left[i\right]$ [2]:

(10) $$G(\pi_{j}):=\frac{1}{N}\sum_{i=1}^{N}G_{j}\left[i\right]$$

(11) $$\begin{array}{cc} G_{j}\left[i\right]:= & G\left(\pi_{j}\left[i\right];Q^{s}\left[t\right]\right):=G\left({\hat{Q}}_{j}^{s}\left[t+i\right]\right):=\\ \; & -\underset{\mathrm{Information}\;\mathrm{Gain}}{\underset{︸}{E_{s∼{\hat{Q}}_{j}^{s}\left[t+i\right];\Sigma_{p}∼Q^{p};\tilde{o}∼N\left(g\left(s\right),\Sigma_{p}\right)}\left[D_{\mathrm{KL}}\left(\mathrm{VI}\left({\hat{Q}}_{j}^{s}\left[t+i\right],\tilde{o}\right)\;∥\;{\hat{Q}}_{j}^{s}\left[t+i\right]\right)\right]}}\\ \; & -\underset{\mathrm{Pragmatic}\;\mathrm{Value}}{\underset{︸}{E_{s∼{\hat{Q}}_{j}^{s}\left[t+i\right];\Sigma_{p}∼Q^{p};\tilde{o}∼N\left(g\left(s\right),\Sigma_{p}\right)}\left[\ln P^{c}\left(\tilde{o}\right)\right]}}. \end{array}$$

The Information Gain is based on an updated belief $\mathrm{VI}\left({\hat{Q}}_{j}^{s}\left[t+i\right],\tilde{o}\right)$ with sampled, ‘hallucinated’, observations $\tilde{o}$. The Pragmatic Value depends on the agent’s preferences, which are given as a distribution $P^{c}$ over observations. Finally, the plan that yields the roll out with the lowest EFE is chosen,

(12) $$\pi^{*}:=\pi_{j^{*}},\;j^{*}:=\underset{j\in \{1,\ldots ,K\}}{\mathrm{argmin}}G\left(\pi_{j}\right).$$

**Figure A1:**
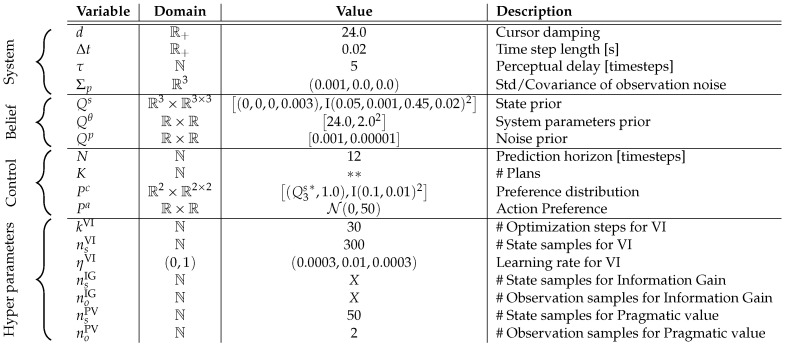
**Table of Parameters**. The value column shows the commonly used value, if not otherwise stated in the paper. *: Value depends on agent’s belief; **: Value is explicitly explained in Section 3.3; X: Value is not used for the results; #: Number of …. For details see Section 3.1.

Algorithm 1 shows pseudo code of the complete planning phase, given the initial belief of the agent $Q^{s}\left[1\right]$ and the parameters in Figure A1.
**Algorithm 1** Planning Phase**Output:** $\pi^{*},G_{j^{*}},{\hat{Q}}^{s}$  **procedure** Planning($Q^{s}\left[t\right]$)        $\pi_{j}\leftarrow {\left(\pi_{j}\left[i\right]\right)}_{ij},\forall i\in \left\{1,\ldots ,N\right\},j\in \left\{1,\ldots ,K\right\};\pi_{j}\left[i\right]∼P_{a}$            ▹ Sample plans        $G_{j}\left[i\right]\leftarrow G\left(\pi_{j}\left[i\right];Q^{s}\left[t\right]\right)$                  ▹ Calculate step-wise Free Energy        $j^{*}\leftarrow \underset{j\in \{1,\ldots ,K\}}{\mathrm{argmin}}\frac{1}{N}\sum_{i=1}^{N}G_{j}\left[i\right]$;$\pi^{*}\leftarrow \pi_{j^{*}}$                   ▹ Select min EFE plan       **for** i∈{1,…,N} **do**                      ▹ Predict future beliefs             ${\hat{Q}}^{s}\left[t+i\right]\leftarrow \mathrm{UKF}\left({\hat{Q}}^{s}\left[t+i-1\right],\pi^{*}\left[i\right];Q^{\theta }\right);{\hat{Q}}^{s}\left[t\right]=Q^{s}\left[t\right]$        **end for**  **end procedure**

### 3.2. Intermittent Active Inference

**Figure 2:**
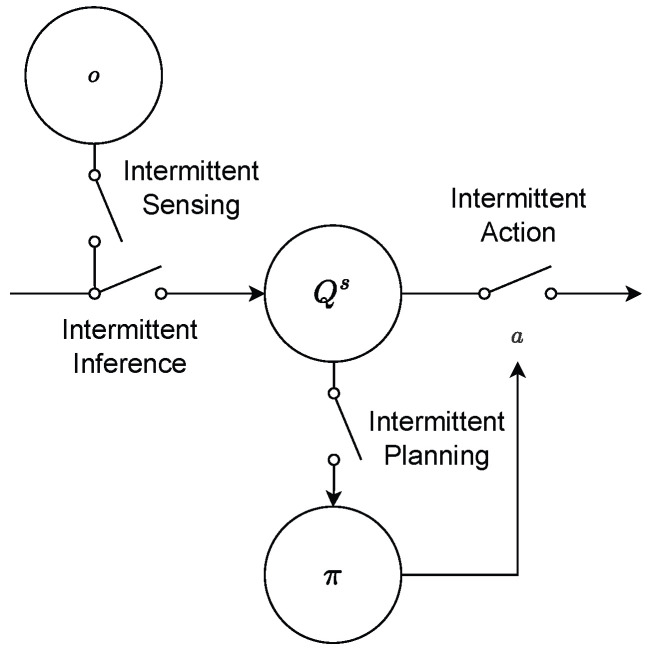
**Different types of IAIF agents**. In standard AIF, all processes are performed in each time step. Since planning is the strongest cause of computation time, in this paper, we focus on agents that sense, infer, and act continuously but plan intermittently. This diagram depicts a repeating building block in the workflow of an IAIF agent, shown in Figures 4 and 5.

The classic processes within an AIF agent are sensing, inference, planning, and action. Traditionally, they are each performed in every single time step. As argued above, reducing this frequency improves computation time and, potentially, the realism in applications such as human motor control. Therefore, we introduce *Intermittent Active Inference* (IAIF) as a term describing AIF agents that sense, infer, plan, or act intermittently, or a combination thereof (see Figure 2). *Intermittent sensing* may be applied in situations when making an observation itself comes with a cost, e.g., the energy required to process visual information in the visual cortex. *Intermittent action* means that the agent may choose to not perform an action, e.g., in situations where actions interfere with sensing or when optimal sensing rates differ from action frequency. *Intermittent inference* allows occasionally skipping the belief update to save computation time, for instance, when the predicted information gain is low.

**Figure 3:**
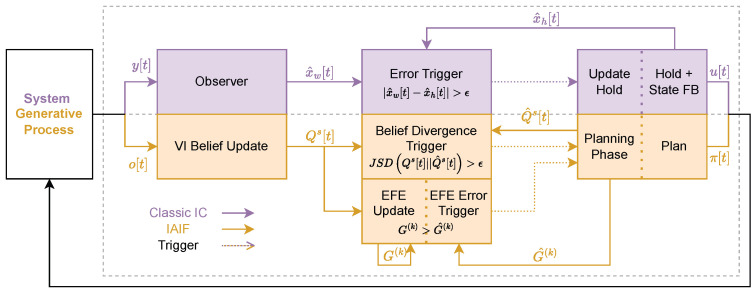
**Control Diagrams for Classic Intermittent Control (purple, upper blocks) and Intermittent Active Inference (orange, lower blocks)**. Both, IC and IAIF start by sampling an observation *y*/*o* from the system or generative process (the diagram shows both processes, but only one of these is applied). This observation is processed by an observer or by the VI belief update, obtaining an updated state ${\hat{x}}_{w}$ or belief $Q^{s}$. Then, the agents decide whether to trigger a hold update or planning phase. For classic IC, this is governed by the prediction error trigger, which compares the updated state with the open-loop hold state ${\hat{x}}_{h}$. For AIF, we propose two different mechanisms: a belief divergence and EFE Error Trigger. The Belief Divergence Trigger compares the belief distribution generated during planning ${\hat{Q}}^{s}$ with the updated belief. The EFE Error Trigger compares an update of the EFE given the new belief *G* with the truncated EFE calculated during planning $\hat{G}$. When triggered (dotted lines), a new hold that generates state feedback (FB) *u* is found (IC), or a new plan π is obtained by minimising the EFE.

*Intermittent planning* offers the strongest lever for reducing computation time, in particular for sample-based AIF. In this initial work, we therefore focus on agents that sense, infer, and act continuously but plan intermittently—for the remainder of this paper that is what we refer to when we use the term *IAIF agent*. An IAIF agent will follow a plan and continuously observe the world, until a re-planning is triggered. This trigger happens when the predictions made during planning were wrong. This is the case when either the agent’s belief about the states diverge significantly from what was predicted during planning or the chosen plan turns out to be worse than expected—for example, if the environment or target has changed unexpectedly. Figure 3 shows a general control diagram that compares classic IC with our proposed approach. The main differences are the deterministic (IC) vs. probabilistic (IAIF) approach and within the triggering mechanisms that determine when to start a new planning phase. In the following, we will introduce practical implementations of a *Belief Divergence Trigger* (Section 3.2.1) and an *Expected Free Energy Error Trigger* (Section 3.2.2). Agents may apply only one of these triggers or a combination to decide when to start a new planning phase.

#### 3.2.1. Belief Divergence Trigger

**Figure 4:**
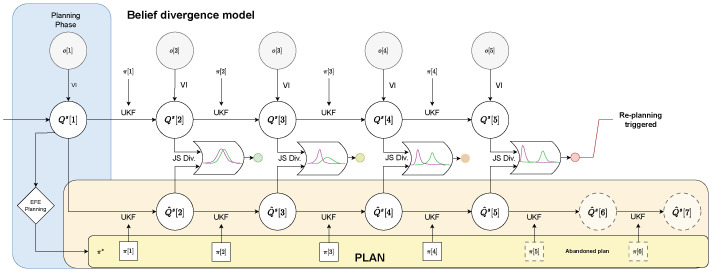
**Flow Chart for the Belief Divergence Trigger Mechanism.** After the planning phase, the agent has chosen a plan $\pi^{*}$ to follow (yellow plan). After applying an action *a*, the agent forwards its belief by applying an unscented Kalman filter (UKF). The agent then receives an observation *o* and updates its belief about the system state $Q^{s}$ using variational inference (VI). In every time step, this updated belief is compared with the agent’s belief for this time step during planning ${\hat{Q}}^{s}$ (orange area). Only when the Jensen-Shannon divergence (JS Div.) surpasses the provided threshold $\epsilon_{\mathrm{Div}}$, the current plan is abandoned and a new planning phase is triggered (step 5).

In classic IC, closing the loop is triggered when the prediction error exceeds a predefined threshold. This error is commonly defined as the distance between predicted state and the state inferred by an observer, which is usually a type of Kalman filter (see Figure 3). In AIF, the agent’s belief about states is described as a probability distribution. This belief is continuously updated, using the agent’s generative model and new observations. The natural translation of the trigger for classic IC is therefore to calculate the difference between the predicted distribution of states during planning and the updated belief after making observations. We make use of the Jensen-Shannon divergence as a measure of the difference between these distributions. Similar to classic IC, if this prediction error surpasses the predefined threshold, a new planning phase is triggered. Otherwise, the agent continues following its current plan. Figure 4 shows the general control flow for an agent that applies this *Belief Divergence Trigger* (Div Trigger).

In detail, the process is described as follows. When starting the simulation, an initial planning phase is required, see Section 3.1.2. The beliefs about the system state generated by rolling out the selected plan (see Equation (9)), ${\hat{Q}}^{s}\left[t+i\right]$ for i∈{1,…,N}, provide a reference of how the agent predicts its belief to change when applying each action along the plan. In each time step *t* the agent applies the next action to the system and receives an observation o[t+1]. The agent updates its belief about the system state $Q^{s}\left[t\right]$ using this information as described in Section 3.1.1. To determine whether a re-planning is triggered, the Jensen-Shannon divergence $D_{\mathrm{JS}}$ between the new posterior $Q^{s}\left[t\right]$ and the predicted belief during planning ${\hat{Q}}^{s}\left[t\right]$ is compared to a predefined threshold $\epsilon_{\mathrm{Div}}>0$. A new planning phase is started if

(13) $$D_{\mathrm{JS}}\left({\hat{Q}}^{s}\left[t\right]\;∥\;Q^{s}\left[t\right]\right)=\frac{1}{2}\left(D_{\mathrm{KL}}\left({\hat{Q}}^{s}\left[t\right]\;∥\;Q^{s}\left[t\right]\right)+D_{\mathrm{KL}}\left(Q^{s}\left[t\right]\;∥\;{\hat{Q}}^{s}\left[t\right]\right)\right)>\epsilon_{\mathrm{Div}}.$$

A mandatory planning phase additionally starts if we run out of plans. The plan and the resulting belief reference ${\hat{Q}}^{s}$ are then updated and the first action is applied. The resulting interaction loop is displayed in Algorithm 2 (‘Div Trigger’ set to ‘True’, ‘EFE Trigger’ set to ‘False’).

It is possible to extend this idea to the agent’s belief about system parameters $Q^{\theta }$ or noise $Q^{p}$. Since these do not change by acting upon the system, it is not necessary to keep track of predictions. Instead the JS distance between the belief during planning and subsequently updated beliefs can be directly used as a measure for ‘learning rate’. If this learning rate exceeds a predefined threshold, it indicates that system parameters or noise differ significantly from what the agent assumed during planning, justifying a new planning phase.
**Algorithm 2** Intermittent Active Inference
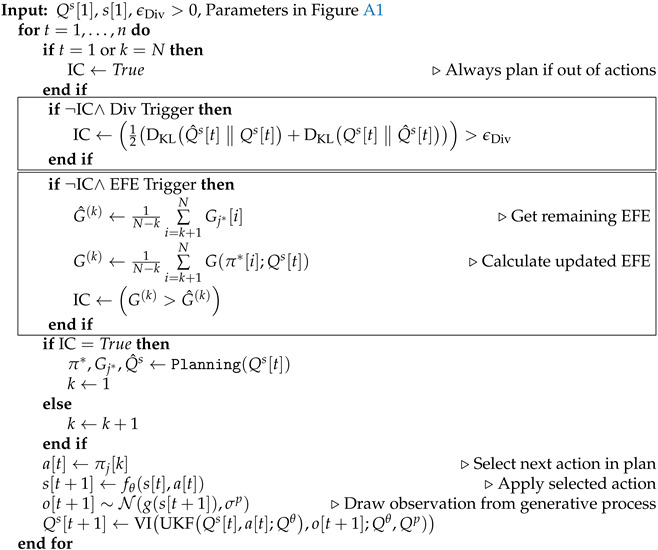


#### 3.2.2. Expected Free Energy Error Trigger

**Figure 5:**
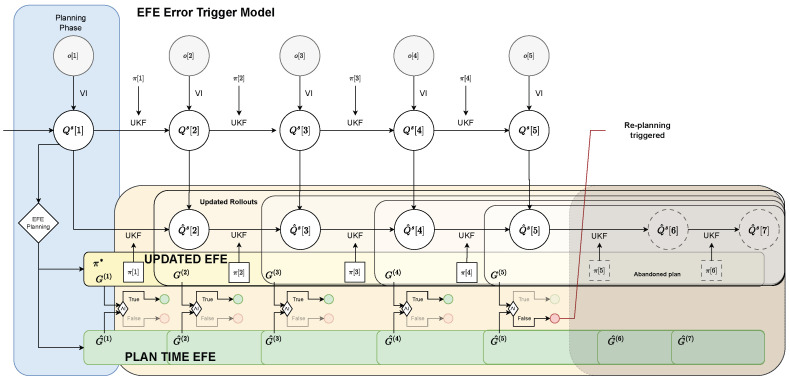
**Flow Chart for the EFE Error Trigger Mechanism.** The general process is similar to Figure 4. Instead of comparing beliefs directly, the agent performs new rollouts for the remaining plan using the novel information (indicated by white areas). These are then used to update the EFE estimate *G* (yellow area). This update is compared with the (truncated) EFE calculated during planning $\hat{G}$ (green area). As long as $G\leq \hat{G}$, the current plan is as good as, or better than expected and is continued. However, when $G>\hat{G}$, the current plan is abandoned and a new planning phase is triggered (step 5).

With the Belief Divergence trigger defined in Section 3.2.1, re-planning is triggered as soon as the agent’s belief deviates enough from the predictions made during planning. However, this might not be necessary as long as new observations point towards equal or even better performance than expected. For example, during planning, the agent may have assumed that things would turn out worse than they ultimately did. Moreover, a re-planning in classic IC can also occur when the reference changes which is typically achieved by including the setpoint in the observed state vector [6]. From a high level AIF perspective, the goal of planning is to choose plans that have the smallest Expected Free Energy (EFE), that is, plans that obtain observations that either are more likely under the agent’s preference distribution (Pragmatic value) or increase salience or novelty (the latter is ignored in this work since we exclude parameter learning for now). (Information gain) (see Equation (11)). We can therefore make use of the EFE calculated during planning and execution to define another trigger for IAIF. During execution of a plan, we utilise the novel information gained from observations to update our EFE prediction for the remainder of the plan. If this prediction is higher than estimated during planning, indicating that the plan became worse, we trigger a new planning phase. The flow chart in Figure 5 provides a schematic representation of the function of the *Expected Free Energy Error Trigger* (EFE Trigger).

In detail, this trigger mechanism works as follows. During planning, we obtain rollouts and step-wise EFEs for all sampled plans (see Section 3.1.2). We keep track of the step-wise EFE for the chosen plan $\pi^{*}$

(14) $$G_{j^{*}}\left[i\right]:=G\left(\pi^{*}\left[i\right];{\hat{Q}}^{s}\left[t\right]\right),i\in \left\{1\ldots ,N\right\}.$$

At each time step k∈{1,…,N} after planning, we apply action $\pi^{*}\left[k\right]$ to the system and obtain a new observation o[t+k] (see Section 3.1.1). We use this information to update our belief about the state to $Q^{s}\left[t+k\right]$. Then, we calculate the (updated) Free Energy as

(15) $$G^{\left(k\right)}=\frac{1}{N-k}\sum_{i=k+1}^{N}G\left(\pi^{*}\left[i\right];Q^{s}\left[t+k\right]\right),$$

by rolling out the remaining plan, starting with the updated belief. Finally, this value is compared with the (truncated) EFE predicted for the same time steps during planning,

(16) $${\hat{G}}^{\left(k\right)}=\frac{1}{N-k}\sum_{i=k+1}^{N}G_{j^{*}}\left[i\right].$$

If $G^{\left(k\right)}\leq {\hat{G}}^{\left(k\right)}$, this means that the plan turned out to be as good as, or even better than, expected and therefore can be maintained. In the other case, i.e., $G^{\left(k\right)}>{\hat{G}}^{\left(k\right)}$, the current plan is no longer as good as previously predicted and is abandoned. A mandatory planning phase additionally starts if we run out of plans, i.e., if k=N. The resulting algorithm is shown in Algorithm 2 (‘Div Trigger’ set to ‘False’, ‘EFE Trigger’ set to ‘True’).

#### 3.2.3. Combined Triggers

In some cases, combining both aforementioned trigger mechanisms leads to the best performance. On the one hand, using only the Belief Divergence Trigger might result in an agent maintaining a plan, although it leads to bad performance. On the other hand, applying the EFE Error Trigger only, an agent might follow a plan that only becomes better than expected because the agent’s belief was poorly aligned with the true state during planning. For instance, before observing where the target is, the agent is likely to choose a plan that moves only roughly in the right direction. Upon perceiving the target, the actual EFE of this plan could improve, since the pragmatic value becomes better by decreasing the agent’s uncertainty about the target. In this case, no new planning phase is started although the new information about the target would allow for a much better plan, which remains unexplored. Adding a Belief Divergence Trigger would cause the agent to re-plan as soon as new observations cause a significant change in the agent’s belief, e.g., observing that the target is somewhere else drastically changes the agent’s belief about the target position. Therefore, we also evaluate agents that combine both trigger mechanisms. The combined version is shown in Algorithm 2 if ‘Div Trigger’ and ‘EFE Trigger’ are set to ‘True’. The Belief Divergence Trigger is always tested first, since we can skip re-estimation of the EFE if the Div Trigger is already met.

### 3.3. Experimental Design

We explore the effects of intermittency on a human user model of mouse pointing. The model is based on the one presented in [11], however we removed the click mechanic to improve the interpretability of the agent’s preference and gain clearer insights on the trigger mechanisms. The goal of the pointing task is to move a 1D mouse cursor into a target area. As performance criterion, we therefore define the percentage of time steps in which the cursor is inside the target, i.e., the time on target. The cursor is modelled as a second-order lag with zero stiffness and a constant damping parameter d>0. The system state consists of the cursor position $s_{1}$ and velocity $s_{2}$, and the target position $s_{3}$ and width $s_{4}$. The agent obtains feedback consisting of the position of the mouse cursor $o_{1}$, a binary value indicating whether the cursor is inside the target ($o_{2}=1$) or not ($o_{2}=0$), the target position $o_{3}$, and target width $o_{4}$. The generative process is therefore given by

$$\begin{array}{cc} \dot{f}\left(s,a\right) & ={\left[s_{2},-ds_{2}+a,0,0\right]}^{T}\\ g(s) & ={\left[s_{1},s_{3},s_{4},c\right]}^{T},\\ c & =\left\{\begin{array}{c} 1,\mathrm{if}\;|s_{1}-s_{3}|\leq s_{4}\;(\mathrm{Cursor}\;\mathrm{inside}\;\mathrm{target})\\ 0,\mathrm{if}\;|s_{1}-s_{3}|>s_{4}\;(\mathrm{Cursor}\;\mathrm{outside}\;\mathrm{target}). \end{array}\right. \end{array}$$

We assume that the agent is trained in the task and we therefore set the generative model equal to the generative process, with some uncertainty about the stiffness parameter *d*. To simulate human perception, we add Gaussian noise and fixed temporal delay of 100 ms. The agent’s preference distribution is defined such that it prefers observing that the cursor is inside the target, as well as being close to the target’s centre. The latter is based on the agent’s belief about the target position, which is only updated after the perceptual delay (the complete IAIF algorithm with perceptual delay is given in Algorithm A1). To reduce side effects during the VI update, we run independent updates for the cursor position and velocity, the target position, and the target width. Since the belief about target position changes drastically when the target is first observed, we also set a higher learning rate for the target position. Additionally, we exclude the computationally expensive information gain during planning since in initial tests we did not observe it having an effect on the agent’s behaviour.

**Figure 6:**
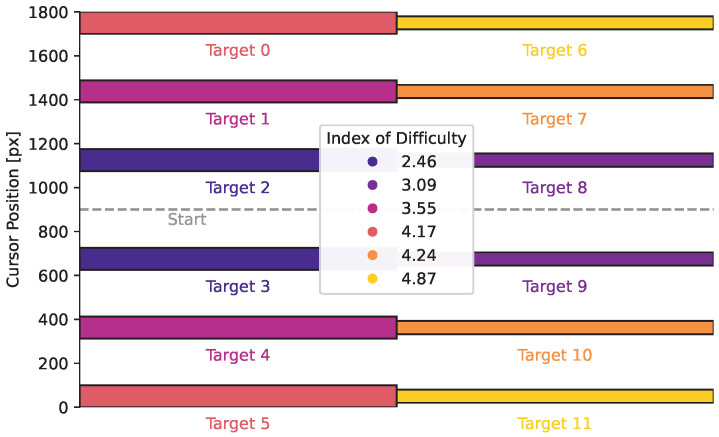
**Target Locations and Widths.** The targets are spread out uniformly, with the cursor always starting in the centre. Targets are either 100 px (targets 0 to 5) or 60 px wide (targets 6 to 11). The legend shows the index of difficulty for the different targets.

We run simulations with six different target locations (three on either side of the start) and two different widths. Figure 6 shows the target setup. The targets include six different difficulties, given by the *index of difficulty* (ID), which is usually given in the Shannon form $\log_{2}\left(1+\frac{D}{W}\right)$, where *D* is the distance to the target, and *W* is the width of the target [50,51]. Targets that are close to the start and large are easy and have low IDs, while small targets that are far from the start are harder and have a high IDs. The initial belief about the target position is centred around the start position with high uncertainty, modelling that the target could appear on either side with varying distance.

As well as a comparison with a classic—non-intermittent—AIF baseline, we also explore the effects of choosing different thresholds for the Belief Divergence Trigger, as well as combinations of both trigger mechanisms. Since the benefit of intermittency also depends on the number of plans sampled during the planning phase, we investigate the performance of agents with various numbers of samples.

### 3.4. Declaration of GenAI Usage

The associated code was partly written with the support of GitHub Copilot (https://github.com/features/copilot, accessed on 5 February 2026), using Claude Sonnet 4.5 (https://www.anthropic.com/claude/sonnet, accessed on 5 February 2026). Every generated line of code was inspected and checked for correctness by the authors. No GenAI was directly involved in the generation, presentation, or interpretation of results.

## 4. Results

In this section we present the results from the simulation study. A complete list of parameters used can be found in Appendix A. Unless mentioned otherwise, in each planning phase 1000 different plans with a horizon of twelve time steps are sampled. Simulations are run for one-hundred time steps with a length of 0.02 s, resulting in a total trial length of two seconds. Targets do not change during one trial, instead, each trial is started from the same initial position with zero velocity and fixed initial uncertainty. For numerical reasons, the position and velocities are scaled down by a factor of 1000 during simulation, however, we present the upscaled results in pixel. We ran ten trials for each of the twelve targets, leading to a total of 120 runs per agent. We ran two-sided Mann-Whitney-Wilcoxon tests to identify significant differences in results. If not mentioned otherwise, box plots contain 120 data points each, the line shows the median, the box 25% (Q1) and 75% (Q3) quantiles, the whiskers show Q1/Q3 minus/plus 1.5 times interquartile range, and any outliers are indicated by circles. Simulations were performed on a machine with a 32-Core AMD Ryzen Threadripper PRO 3975WX CPU, 512GiB DDR-4 RAM, and using a single NVIDIA GeForce RTX 3090. The main parts of the code are optimised using the hardware-accelerated version of JAX (https://docs.jax.dev/en/latest/index.html, accessed on 5 February 2026).

### 4.1. Analysis of the Impact of Intermittency in Agent Behaviour

**Figure 7:**
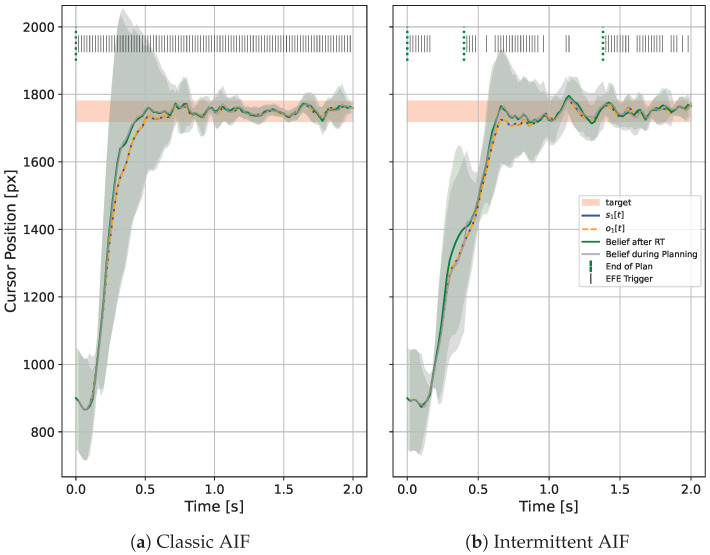
**Mouse Cursor Trajectory Simulated with Classic and Intermittent Active Inference for a 1D Pointing Task.** The blue and orange lines show the true cursor position *x* and the observation *o*, the mean of the agent’s prediction is shown as a green line, with 3σ shown as area. The target is displayed as a red area. (**a**) A classic AIF agent observes and plans at every time step. (**b**) The intermittent AIF agent only starts a planning phase when the expected free energy of the current plan turns out to be worse than predicted during planning. These trigger events are displayed by dashed red lines. The agent’s predictions of how the cursor will behave following its plan is displayed as a gray line and area. During the onset of the movement, the agent’s uncertainty about the cursor position is large due to a fast movement towards the target. This results in many trigger events. As soon as the cursor reached the target and the uncertainty collapses, the agent’s plan turns out to be better than expected ($e_{\mathrm{EFE}}<1$ after ≈0.7 s) and it confidently follows its plan. In this phase, the agent only plans intermittently, but achieves equal performance.

The qualitative behaviour between classic and intermittent AIF in individual trials can differ. Figure 7 shows the cursor trajectory of a movement to target 6 for classic AIF and IAIF. While the classic AIF agent moves towards the target as soon as it is observed (after 0.1 s), the IAIF agent keeps following a plan that slows down halfway through the surge phase (0.3 s). It only re-plans when the plan terminates (indicated by a green line on top of the plot). This is reasonable, since the plan still results in a movement towards the target and there is no risk of overshooting the target. When getting closer to the target, re-planning is triggered more frequently until the agent is confident that the cursor stays on target. At around 1.1 s, the cursor is moved out of the target by mistake, which triggers another planning phase. Interestingly, this recreates human-like variability as seen in [34].

**Figure 8:**
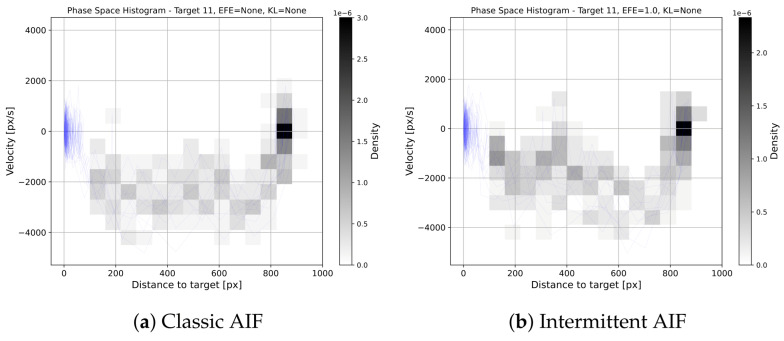
**Phase Space Histograms for Baseline vs. AIF.** The blue lines show the phase space for all 10 trials. The histograms show the density of points in the phase space plots. We excluded the target area (distance <100 px) since many points fall into that area, skewing the histogram. The general shape of the phase spaces is similar, showing similar variance in movements. However, an additional submovement can be noticed that occurs occasionally in the IAIF agent, between 400 px and 450 px distance to target. This submovement can also be found in Figure 7b during the surge phase.

The phasespace histogram in Figure 8 shows the behaviour for target 11, a small target that is far away. The overall shapes of the phase spaces are similar. Introducing intermittency is therefore not increasing the overall variance significantly. However, one can observe an additional submovement that occurs occasionally in the IAIF agent, between 300 px and 400 px distance to target. The individual trajectory discussed above shows a similar submovement, indicating that this behaviour stems from the less frequent planning during the surge phase. This behaviour can also be seen for targets 0, 1, 5, and 6 (all other phasespace histograms are provided in Appendix C). This aligns with the theory that humans perform pointing tasks in multiple corrective submovements [29].

### 4.2. Intermittency Shows No Negative Impact on Performance

**Figure 9:**
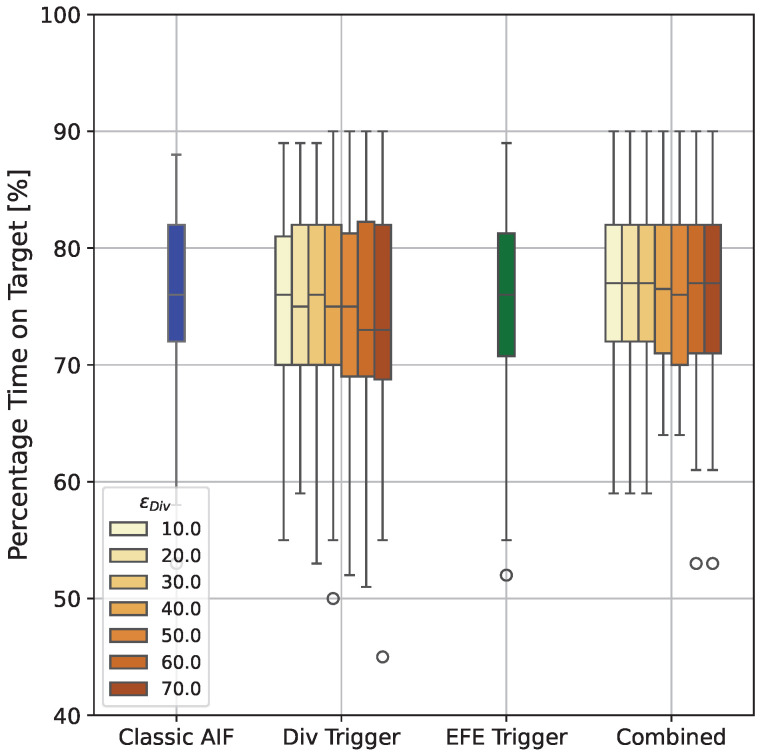
**Percentage Time on Target for all Agents.** The first box shows the performance of the classic AIF agent, the second group shows agents with Belief Divergence Trigger only, the third group shows the agent with EFE Error Trigger only, and the last group shows agents with both triggers combined. Colours indicate the error threshold for the employed Belief Divergence Trigger. No agent shows a significant difference in performance. Agents that only apply the Belief Divergence Trigger with large threshold can have slightly worse mean performance, while all other agents achieve similar performance as classic AIF.

Figure 9 shows the percentage time on target for all explored agents. Over all simulated trials and for all IAIF agents, the performance of IAIF agents does not significantly differ from the classic AIF agent. The only visible effect is that compared to the baseline, using the Belief Divergence Trigger (second group from the left) with a threshold which is too high, can lead to slightly lower mean performance (Classic AIF: 76.4%, AIF with Div trigger only: 74.7% [$\epsilon_{\mathrm{Div}}=50.0$], 74.2% [$\epsilon_{\mathrm{Div}}=60.0$], 74.3% [$\epsilon_{\mathrm{Div}}=70.0$]). Looking at individual trials, the reason for this is that there are moments where the cursor is already inside the target and the agent keeps following a plan that moves it outside the target area again. This can happen because of poor sampling during planning and low uncertainty, preventing the agent from starting a new planning phase. The agent applying the EFE Trigger only (third group) achieve similar performance (75.4%) to the Baseline. Same holds for the agents using both triggers (forth group, all means between 76.3 and 76.9 for the agent with $\epsilon_{\mathrm{Div}}=30.0$).

### 4.3. Intermittent Active Inference Plans Less and Saves Computation Time

**Figure 10:**
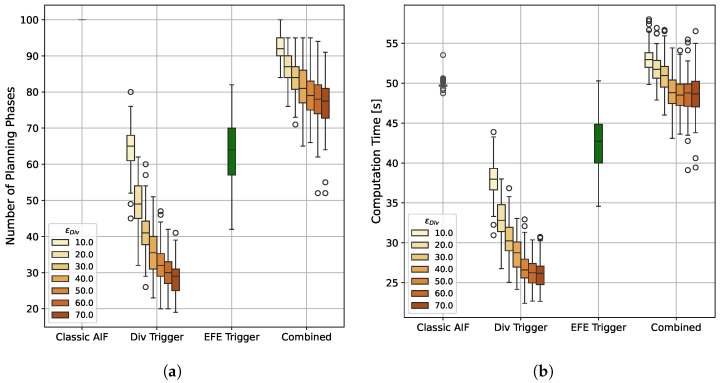
**Number of Planning Phases and Computation Time per Trial.** The first box shows the classic AIF agent, the second group shows agents that only apply the Belief Divergence Trigger with different thresholds $\epsilon_{\mathrm{Div}}$, the third group shows the agent that only applies the EFE Error Trigger, and the last group shows agents that apply both triggers with different Div Trigger thresholds. (**a**) While the standard AIF baseline plans in all 100 time steps, all IAIF agents plan less often. The agents applying the Div Trigger only require the least number of re-plannings, which also decreases significantly with higher thresholds. The agent that only applies the EFE Trigger requires an average of 64.1 planning phases, meaning that re-planning effort can be reduced by 35.9%. (**b**) The baseline consistently takes around 50 s per trial to compute. Agents with Div Trigger only have the quickest computation time, with the fastest agent only taking 26.1 s in average. The agent that only applies the EFE Trigger can reduce the computation time to an average of 42.6 s. Agents with combined triggers have higher computation time which can be higher than classic AIF due to the overhead introduced by EFE re-estimation.

The main driver of computation time for continuous AIF is the sample-based planning. Using intermittent planning, we are able to reduce the number of time steps in which a planning phase is performed significantly, see Figure 10a. The baseline AIF agent selects a new plan in each of the one-hundred time steps, leading to a mean per trial computation time of 49.8 s. In contrast, agents with Belief Divergence Trigger only plan between 28.4 and 64.5 times on average, reducing the mean per-trial computation time to 26.1 s and 38.0 s (shown in Figure 10, second group). The agent that only applies the EFE Trigger uses an average of 64.1 planning phases (Figure 10a third group). However, to calculate the EFE, an additional rollout is necessary in each time step (see Equation (15)), which has a negative impact on computation time. Despite this overhead, the EFE error only agent manages to significantly reduce the mean computation time by 14.5% to 42.6 s (Figure 10b third group). Agents that combine both triggers show more frequent re-planning and also higher computation times that can even surpass the classic AIF (see Figure 10, right group).

**Figure 11:**
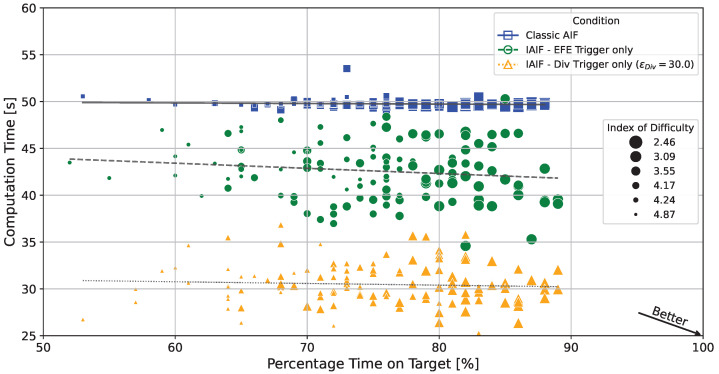
**Percentage Time on Target vs. Computation Time for Individual Trials.** Each point corresponds to one individual trial. The lines show linear regressions for the different agents. Trials in the lower right strike the best trade-off, while points in the upper left correspond to weak trials. The classic AIF agent (blue squares/solid lines) consistently takes around 49 s and 50 s per trial, with time on target between 60% and 90%. Both shown IAIF agents (only EFE Trigger, green circles/dashed lines; only Div Trigger with $\epsilon_{\mathrm{Div}}=30.0$, orange triangles/dotted lines) have significantly reduced computation times, while maintaining similar time on target. The size shows the index of difficulty of the target (the smaller the marker, the higher the difficulty). While easier targets (lower ID) consistently show higher time on target, computation time is mostly independent of task difficulty.

If error thresholds are chosen correctly, agents that plan intermittently can achieve similar task performance while significantly reducing computation time. Figure 11 shows the computation time and performance of all simulated trials for the classic AIF baseline (crosses/solid line), an IAIF agent with only EFE Error Trigger (circles/dashed line), and an IAIF agent with only Belief Divergence Trigger with $\epsilon_{\mathrm{Div}}=30.0$ (triangles/dotted line). All individual IAIF trials have a lower computation time than classic AIF. Simultaneously, they achieve similar percentage time on target between 60 and 90, with only a few outliers. There is no clear trend showing that higher computation time leads to better performance. Easier targets (ID 2.46, 3.09, 3.55) show higher percentage time on target for all agents. These results indicate that intermittency can be introduced into AIF without compromising performance.

### 4.4. Analysis of the Effect of Number of Sampled Plans on Classic and Intermittent Active Inference

**Figure 12:**
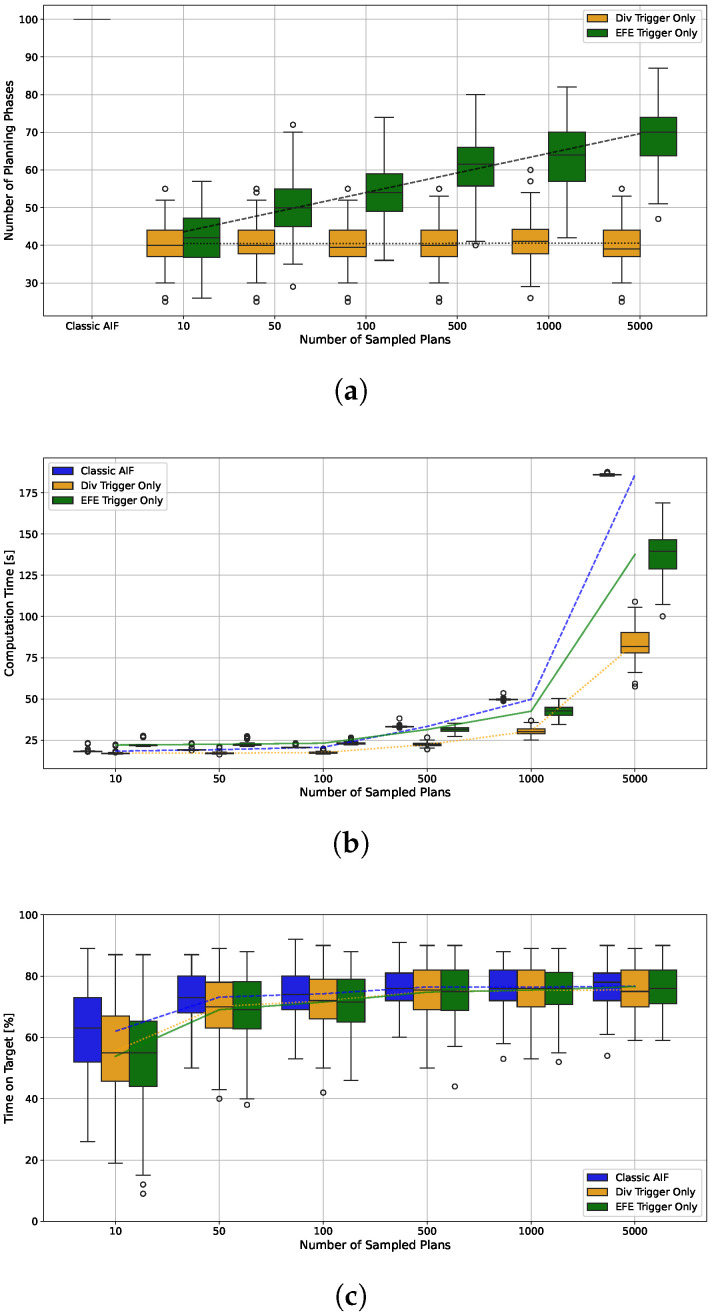
**(a) Number of Planning Phases (b) Computation Time per Trial (c) Percentage Time on Target for Different Numbers of Sampled Plans *K*.** The box plots contain 120 trials each, in line with the previous results. Lines show the development of the means. (**a**) For the agent with EFE Error Trigger only (green) the number of planning phases increases logarithmically in the number of sampled plans. The agent with Belief Divergence Trigger only is not significantly affected. (**b**) The computation time increases in the number of sampled plans. The agent with Div Trigger has the lowest computation time for all number of plans. The overhead introduced through extra roll-outs for the EFE Trigger result in a higher computation time then classic AIF for 10, 50, and 100 sampled plans. However, IAIF is faster than the baseline when 500 or more plans are sampled, since the computation time of planning surpasses the overhead, which is independent of *K*. (**c**) For all agents, the performance is lower if *K*, is small. For larger *K*, the difference in performance of classic and intermittent AIF becomes smaller.

The EFE Error Trigger introduces an additional overhead for re-estimating the EFE in every time step (see Equation (15)). This overhead is independent on the number of plans K∈N sampled during planning since only the current plan is rolled out (and only depends on the length of the remaining planning horizon). We therefore expect an increased benefit of intermittency when *K* is large. We explore this effect for three different agents, the classic AIF baseline, an agent with Belief Divergence Trigger only and $\epsilon_{\mathrm{Div}}=30.0$, and an agent with EFE Error Trigger only. Figure 12a shows that with higher *K*, the number of planning phases for the agent with EFE Trigger increases, while it does not have an effect on the agent with Div Trigger. The computation time displayed in Figure 12b shows a clear trend: Using the Div Trigger only leads to the lowest computation time for all different *K*. For agents with low values of *K*, the classic AIF is faster than the EFE Trigger agent despite having to plan in every time step. This changes for K≥500, where IAIF becomes faster than the baseline. The trend indicates that the relative time saving increases further with higher *K*. For the largest tested K=5000, the mean computation time can be reduced from 185.9 s to 137.6 s (EFE Trigger) and 83.6 s (Div Trigger) respectively. The performance shows a similar trend, see Figure 12c. IAIF agents perform worse than the classic AIF agent only for K≤500. All agents show similar variance in performance, which is larger if less plans are sampled. Since larger values for *K* become necessary for more difficult problems and actions with higher dimension, these results indicate that using intermittency may become very beneficial—if not necessary—for applications with constrained computational resources.

## 5. Discussion and Future Work

### 5.1. Guidelines on Applying Intermittent Active Inference

In this paper, we have explored Intermittent Active Inference as an extension to classic sample-based Active Inference. Following the results, agents that apply intermittent planning using either only the Belief Divergence Trigger with thresholds lower than 50, or agents using only EFE Error Trigger achieve similar performance to the baseline, while simultaneously cutting planning phases and computation time. The Div Trigger offers an approach which can reduce the computation time even further, however choosing the correct threshold $\epsilon_{\mathrm{Div}}$ depends on the problem at hand and requires thoughtful consideration, since choosing a too high threshold can deteriorate performance. Instead, the threshold could be treated as a hyperparameter that can be learned based on previous interactions, requiring repeated trials in a variety of related scenarios. The threshold with the best trade-off between computation time and performance is likely to strongly depend on the situation (environment, agent and task/preference prior). For instance, in high risk scenarios, choosing a smaller threshold is appropriate despite the computational effort. Since combining both triggers did not significantly improve the performance but increased computation time (and comes with the same issue of finding the correct threshold), we recommend either using only the EFE Error Trigger or only Belief Divergence Trigger. Ultimately, the EFE Trigger is based on the Free Energy Principle itself, which suggests that it is robust and works for a wide range of applications without necessary tuning.

### 5.2. *k*-Step Expected Free Energy Error Trigger

To decrease the impact of the overhead associated with using the EFE Trigger, a *k*-step EFE could be used where only the mean over the next k<N step-wise EFEs is compared. The number of considered steps may also be a learning parameter which depends on the complexity of the situation (when no critical situations are expected, a smaller *k* might suffice). In this context, a 0-step EFE trigger could be implemented by only comparing the Free Energy of the current time step with the estimation made during planning. Although computationally superior, this approach would require a measure of divergence which needs further investigation.

### 5.3. Noise Sensitive Expected Free Energy Error

Since the calculation of EFE is based on samples, the resulting value is subject to noise. The EFE Error Trigger however strictly checks whether the updated EFE is larger than the EFE predicted during planning (see Section 3.2.2). Although this can lead to triggering a planning phase even if the deviation is not significant, this does not pose a large issue in practice. However, to still allow for some deviations, one may set an additional threshold $\epsilon_{EFE}>0$ and only trigger a planning phase if the re-estimated EFE is larger than the predicted EFE during planning plus the threshold, i.e., $G^{\left(k\right)}>{\hat{G}}^{\left(k\right)}+e_{\mathrm{EFE}}$. Since the absolute values of EFE can vary greatly, a threshold on the relative error may be preferred.

### 5.4. Plan Exhaustion and Augmentation

During intermittent periods when no planning phase is triggered, our proposed agent simply follows its current plan, which therefore gradually has a shorter prediction horizon. This can eventually lead to ‘plan exhaustion’, i.e., when the agent runs out of actions and needs to perform a full re-planning. The prediction of EFE for the remainder of the plan also becomes less reliable, since it does not take time steps after the remaining planning horizon into account, potentially missing undesirable outcomes. Instead, one could explore different heuristics to augment the agent’s current plan, such as appending new actions.

### 5.5. Minimum and Maximum Re-Planning Intervals

Following the theory of a psychological refractory period [52], in addition to intermittency, a minimum re-planning interval can be introduced. A refractory period that is at least as large as the perceptual delay is part of the conventional formulation of IC for computational reasons [6], because re-planning makes no sense if we have not yet observed the impact of our first actions. This ensures that the agent will always follow a new plan for the first few time steps until the end of this interval. It can mitigate issues with system identification commonly observed in closed-loop systems with observation noise [45]. Similarly, a maximum re-planning interval chosen between the minimum interval and prediction horizon, could help to reduce negative effects of the shortening prediction horizon, as discussed in Section 5.4.

### 5.6. Intermittency and Prediction Horizons

Within intermittent planning, one could potentially couch adaptive prediction horizon length methods as a form of intermittency, where planning can be aborted given some condition. See e.g., [53,54] for examples of this from model-based RL and MPC. This approach could allow the agent to use short horizons in complex or uncertain, high-model-error regions and longer horizons in stable regions, improving performance, efficiency, and stability.

### 5.7. Intermittent Active Inference for Complex Tasks

In this work, we evaluated IAIF against classic AIF on a simple 1D mouse pointing task. Since intermittency can have different effects depending on the difficulty of a task, future research should explore IAIF for more complex and diverse tasks. In multi-stage tasks that require multiple time steps to achieve a goal (for example, mouse point and click [11]), sample-based continuous re-planning could lead to agents that take long to achieve the second task. This is due to the fact that the best sampled plans may only achieve the second task on the second action in the plan, which is never applied. Here, IAIF may proof to even perform better than classic AIF. We also decided to fix the planning horizon to 12 time steps (or 240 ms) throughout all simulations. While for very short planning horizons the benefit of intermittent planning recedes, longer horizons can be more beneficial since the agent could follow a well-performing plan for longer. However, a longer horizon will also increase the overhead of re-computing the EFE.

### 5.8. Intermittency in Discrete Active Inference

In this paper, we investigate problems with continuous state, observation, and action spaces. We motivate this by the usually higher computational cost involved in sample-based approaches necessary for non-discrete AIF. However, we do not anticipate any limitations for transferring the presented concepts onto discrete problems. In particular, for large scale generative models, in which exploring all possible states is intractable, planning intermittently can have similar benefits. Intermittency can be easily added to existing implementations of AIF by adding a decision on when to infer a new policy, based on one of the triggers suggested in this work (for instance, in pymdp (https://github.com/infer-actively/pymdp, accessed on 5 February 2026), only running infer_policies when a trigger happens, e.g., when the EFE becomes worse than predicted during the last execution).

### 5.9. Role of Intermittency in Active Inference Modelling Practice

The heuristics proposed in this work for intermittent planning can bring several benefits in the early stages of model refinement. (1) By freeing computational budget for speeding up exploration and enabling model improvements, (2) providing the basis for machine learning of context-sensitive re-planning patterns, and (3) informing human design decisions.

(1) The computational benefits of intermittent planning can be used to both speed up simulations of any given agent, but also potentially improve the performance of the agent, for a given computational budget. The savings to the computational budget could be used to broaden sampling, support more complex models, or extend prediction horizons.
(2) The intermittency heuristics can provide the basis for automatic machine learning of context-sensitive patterns. Learning the relationship between context and re-planning can amortise the computation of the metrics, bringing additional benefits in computational savings. Developing this further, if a generative model captures this relationship, the intermittent switches in Figure 2 could be treated as actions of the standard AIF agent, providing a more elegant principled approach to the inclusion of intermittency (the long-term goal would still be to reduce the mismatch between generative model and environment, but at any point in this learning process, intermittency could be used to manage the current state of the model mismatch).
(3) Human designers and modellers can gain insight from (learned) patterns of intermittency to inform model structure development. For instance, context-sensitive rates of intermittency as the agent engages with different aspects of the environment, or different tasks, could indicate that the generative model is struggling to adequately predict behaviour (similar to a Geiger counter). More systematic patterns of intermittent behaviour, such as observing more frequently than acting, or acting more frequently than re-planning, in a flat agent could suggest that a hierarchical model—with updates made at different rates at different hierarchical levels—might be more appropriate for the task.

## 6. Conclusions

We introduced Intermittent Active Inference as an extension of classic Active Inference, focussing on agents that observe, infer, and act continuously, but plan intermittently. The motivation behind intermittency can be attributed to its computational efficiency, its capacity to model similar refractory structures as natural agents, its advantages in closed-loop parameter identification, and its benefits as a practical tool for model refinement. We proposed two mechanisms to trigger re-planning, a Belief Divergence Trigger and an Expected Free Energy Error Trigger, and evaluated standard and Intermittent Active Inference agents on a simple model of a 1D mouse pointing task. Our results indicate that intermittent planning reduces computation time, and while not changing task performance significantly, added some behavioural variability reminiscent of that in human motor control. Furthermore, we showed that significant computation time savings could be made as the number of sampled plans increased. We believe that this simple augmentation of an Active Inference implementation can have immediate practical benefits, is likely to support model development and testing, and that the behavioural effects of intermittency will overlap with those of alternative hierarchical models.

## Figures and Tables

**Figure A2:**
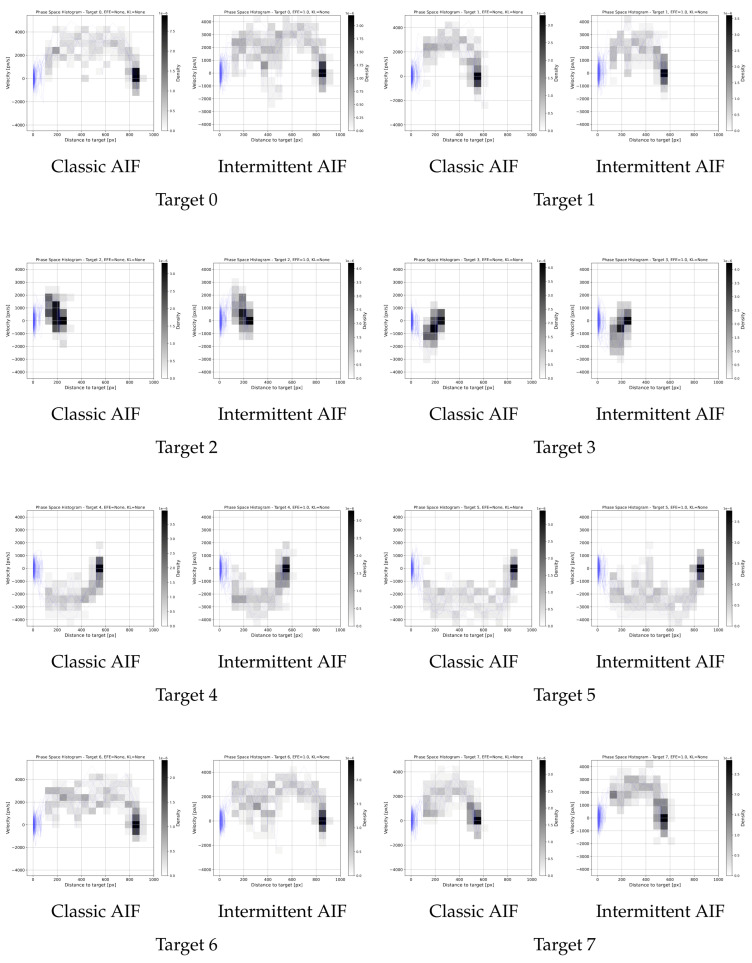

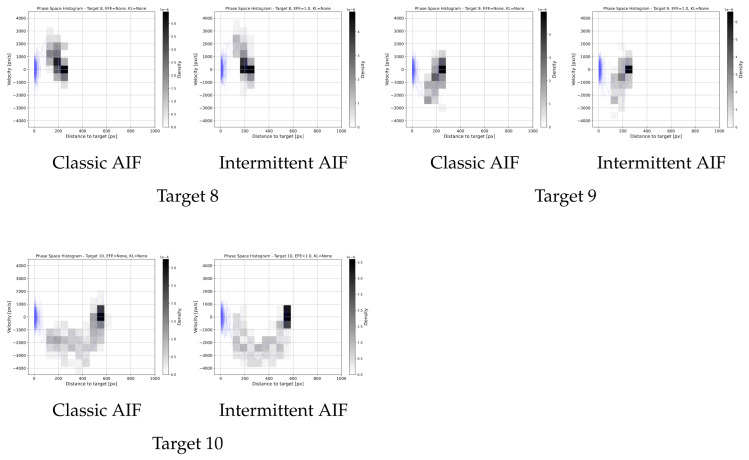
**Phase Space Histograms for Classic** **and Intermittent Active Inference.**

## Data Availability

The data presented in this study are openly available in [Intermittent Active Inference] https://github.com/mkl4r/iaif, accessed on 5 February 2026.

## Appendix A. Parameters

The additional parameters used for the simulation are reported in Figure A1. I(x) stands for the diagonal matrix with *x* on the diagonal.

## Appendix B. Intermittent Active Inference with Perceptual Delay

**Algorithm A1** Intermittent Active Inference with Perceptual Delay

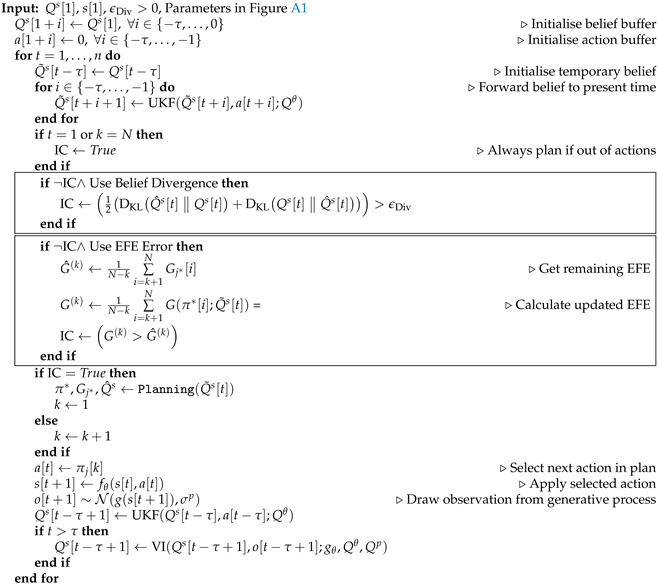

## Appendix C. Additional Results

### Phasespace Histograms

The following plots show the phasespace histograms for the remaining targets.
